# Quercetin Attenuates Vascular Calcification through Suppressed Oxidative Stress in Adenine-Induced Chronic Renal Failure Rats

**DOI:** 10.1155/2017/5716204

**Published:** 2017-06-11

**Authors:** Xue-ying Chang, Lei Cui, Xing-zhi Wang, Lei Zhang, Dan Zhu, Xiao-rong Zhou, Li-rong Hao

**Affiliations:** ^1^Department of Nephrology, The First Affiliated Hospital of Harbin Medical University, Harbin, China; ^2^Department of Occupational Health, Public Health College, Harbin Medical University, Harbin, China

## Abstract

**Background:**

This study investigated whether quercetin could alleviate vascular calcification in experimental chronic renal failure rats induced by adenine.

**Methods:**

32 adult male Wistar rats were randomly divided into 4 groups fed normal diet, normal diet with quercetin supplementation (25 mg/kg·BW/d), 0.75% adenine diet, or adenine diet with quercetin supplementation. All rats were sacrificed after 6 weeks of intervention. Serum renal functions biomarkers and oxidative stress biomarkers were measured and status of vascular calcification in aorta was assessed. Furthermore, the induced nitric oxide synthase (iNOS)/p38 mitogen activated protein kinase (p38MAPK) pathway was determined to explore the potential mechanism.

**Results:**

Adenine successfully induced renal failure and vascular calcification in rat model. Quercetin supplementation reversed unfavorable changes of phosphorous, uric acid (UA) and creatinine levels, malonaldehyde (MDA) content, and superoxide dismutase (SOD) activity in serum and the increases of calcium and alkaline phosphatase (ALP) activity in the aorta (*P* < 0.05) and attenuated calcification and calcium accumulation in the medial layer of vasculature in histopathology. Western blot analysis showed that iNOS/p38MAPK pathway was normalized by the quercetin supplementation.

**Conclusions:**

Quercetin exerted a protective effect on vascular calcification in adenine-induced chronic renal failure rats, possibly through the modulation of oxidative stress and iNOs/p38MAPK pathway.

## 1. Background

Chronic kidney disease (CKD) is defined by reduced glomerular filtration rate or kidney damage, which affects 10–15% of residents in the US, Europe, and Asia [1]. Vascular calcification (VC) is a serious and increasingly prevalent clinical problem, which often happens with advancing age, atherosclerosis, diabetes mellitus, and end-stage renal disease. Mineral metabolism disorder, chronic inflammation, and oxidative stress were considered to contribute to this pathological condition [2]. The impairment in vessel elasticity and compliance of the vessel wall lead to higher risk for myocardial infarction, hypertension, and plaque rupture during angioplasty. VC is also significantly and independently positively correlated with cardiovascular morbidity or mortality in CKD and end-stage renal failure patients. However, few studies have found the efficient substance to reverse or decrease the progression of VC.

Quercetin is an important dietary flavonoid, which is present in red onions, apples, berries, citrus fruits, tea, red wine, and many Chinese herbs [3]. Numerous studies have shown that quercetin shows antioxidant, anti-inflammatory, anticancer, immunomodulatory, antiaggregatory, and vasodilating effects [4, 5] and has been confirmed with safety and effectiveness for cancer [3, 6] and being easy to extract [7, 8]. One recent meta-analysis of 7 trials showed a statistically significant effect of quercetin supplementation in the reduction of blood pressure [9]. Most of the beneficial effects of quercetin are related to its antioxidative properties [3, 8]. Quercetin pretreatment effectively ameliorates LPS-induced acute lung injury, largely through suppression of inflammation and oxidative stress [10]. Quercetin can scavenge free radical and regulate some critical proteins from intracellular signaling cascades. Quercetin shows higher ability of protection of neurons from oxidative stress than that of vitamin C, with favorable effects on the spinal cord by suppressing the activation of p38MAPK/iNOS signaling pathway and attenuating the oxidative stress in the rats with spinal cord injury [11]. In chronic renal failure (CRF) patients, VC is a real challenge for management of CRF [12]. Recent studies have shown that quercetin effectively attenuated streptozocin (STZ) induced cytotoxicity [13] and valproic acid induced oxidative stress in renal tissue [14], which indicates the potential renoprotective effects of quercetin on VC.

To explore more detailed evidence for the protective effects of quercetin on chronic renal failure, we have designed this study to investigate the effects of quercetin on the development of VC in adenine-induced chronic renal failure rats.

## 2. Methods

### 2.1. Animal Treatment

All experimental procedures meet all applicable standards for the ethics of experimentation and research integrity and are approved by the Animal Experimentation Ethics Committee of Harbin Medical University. The chronic renal failure model was prepared as described [15]. Briefly, seven-week-old male Wistar rats (180–220 g) were purchased from Vital River Laboratory (Beijing, China) and completely randomized into 4 groups for treatment by table of random number after one week of acclimatization: group A: normal diet with distilled water administered by gavage; group B: normal diet with quercetin supplementation by gavage (25 mg/kg·BW/d); group C: 0.75% (w/w) adenine diet with distilled water administered by gavage; group D: 0.75% (w/w) adenine diet with quercetin supplementation by gavage (25 mg/kg·BW/d). The normal diet was based on the AIN-93M diet and the adenine diet was the AIN-93M diet with 0.75% adenine added, respectively. The rats were individually housed in an environmentally controlled room at 22 ± 2°C, 50 ± 5% humidity and 12 : 12 h light : dark cycle with free access to food and water.

At the end of week 6, all rats were sacrificed by bloodletting after an overnight fast and pentobarbital anaesthesia. Blood samples were obtained from the abdominal aorta and centrifuged (3000 rpm for 15 min). Serum was then obtained, aliquoted, and stored at* −*80°C until serum analysis within a short period of time. Moreover, thoracic and abdominal arteries of the rats were obtained and flushed with cold phosphate-buffered saline (PBS). Aorta arch was fixed with 10% phosphate-buffered neutral formalin (pH 7.4, 0.1 mol/L) for histology and the remaining artery tissue was used for other experiments (thoracic arteries were used for measurement of INOS, p38MAPK, and p-p38MAPK expressions and abdominal arteries were used for calcium and ALP activity measurement).

### 2.2. Serum Parameter Analysis

The serum uric acid (UA) and creatinine (CRE) were determined by a ROCHE Modular P800 Automatic Biochemical Analyzer (Roche Diagnostics, Mannheim, Germany). Serum superoxide dismutase (SOD), malonaldehyde (MDA), and glutathione peroxidase (GSH-Px) were detected by commercial kits using enzymatic methods (Jiancheng Technology, Nanjing, China).

### 2.3. Calcium and ALP Activity Measurement in Aorta

Calcium content in the aorta was measured as previously described [16]. Briefly, about 10 mg aortic tissue was dissolved in HNO_3_ and diluted with a blank solution (27 nmol/L KCl, 27 mmol/L LaCl_3_). The calcium was determined by atomic absorption spectrophotometry at 422.7 nm (novAA 300; Analytik, Jena AG, Germany). For the alkaline phosphatase (ALP) activity, about 10 mg (1 cm) abdominal aortic tissue was incubated with isotonic PBS (1 : 10, w/v) to prepare tissue homogenates. After centrifugation for 10 min at 4°C, 1600*g*, the supernatant was collected and ALP activity was determined by the use of an ALP detection kit (Jiancheng Technology, Nanjing, China).

### 2.4. Staining of the Aorta

Von Kossa calcium staining was applied to determine calcium in aorta. A 1 cm segment of aorta was excised and fixed with 10% phosphate-buffered neutral formalin (pH 7.4, 0.1 mol/L) for 24 h; then samples were dehydrated in a graded series of ethanol solution and embedded in paraffin [16]. Sections of aortic tissue, 6 mm thick, were cut, and one slide was stained with hematoxylin and eosin. Other slides were deparaffinized, dehydrated, and then treated with 5% AgNO_3_ for 30 min. Specimens were then counterstained with safranin (red staining) and examined under a light microscope.

For the hematein-eosin (HE) staining, aortic tissues were placed in formalin for no more than 24 h and then fixed in neutral-buffered formalin solution, dehydrated in graded alcohol, cleared in xylene, and embedded in paraffin. Then, these blocks were sectioned using a microtome, dehydrated in graded alcohol, embedded in paraffin, sectioned, and stained with hematoxylin and eosin for microscopic examination.

### 2.5. Protein Measurement and Western Blotting

INOS, p38MAPK, and p-p38MAPK expressions of aorta were determined by Western blot analysis. Antibodies against iNOS, p38MAPK, and p-p38MAPK were purchased from Cell Signaling (Beverly, MA, USA). Protein from aorta was extracted with a RIPA lysis buffer, and nuclear protein extracts and total protein extracts were prepared using protein extraction kits (Biotype Institute of Biotechnology). Protein concentrations were determined by the Bradford method (Kruger, 1994). Equal amounts of protein were separated by sodium dodecyl sulfate-polyacrylamide gel electrophoresis (SDS-PAGE) and electrotransferred onto polyvinylidene fluoride (PVDF) membranes. The corresponding molecular weight zone of the membranes was blocked with 5% non-fat milk in Tris-buffered saline containing 0.2% Tween 20 for 30 min and then incubated with primary antibody at 4°C overnight and then completely rinsed and incubated with HRP-conjugated secondary antibody at a constant temperature for 2 h. The signal was amplified by color development using the ProtoBlot II AP System with a stabilized substrate (Promega Corporation, Madison WI, USA). Data are presented as the relative intensity of the protein bands. Experiments were replicated at least three times, and representative blots are shown in the figures. The ratio of iNOS to *β*-actin band intensities and p-p38MAPK to p38MAPK band intensities represented the iNOS or p-p38MAPK protein relative content, respectively, while *β*-actin or p38MAPK served as the internal reference.

### 2.6. Statistical Analysis

All data are expressed as mean ± standard deviation (SD). Data were analyzed using one-way ANOVA followed by post hoc test of LSD, with *P* < 0.05 considered to be significant. All *P* values were two-sided. Statistical analysis was carried out using the SPSS software (version 13; Beijing Stats Data Mining).

## 3. Results

### 3.1. Effects of Quercetin on Serum Calcium, Phosphorous, Kidney Functional Markers, and Oxidative Stress Markers of Chronic Renal Failure Rats

Table 1 illustrates that serum phosphorous, UA, CRE, and MDA concentration were elevated significantly in the adenine diet group. Serum SOD were significantly decreased in the adenine diet group (All *P* < 0.05). Treatment with quercetin significantly prevented the increase of phosphorous, UA, and CRE levels and improved status of inflammation compared to those in the adenine diet group (All *P* < 0.05). However, the level of serum calcium and GSH-Px was not altered.

### 3.2. Effects of Quercetin on Calcium Accumulation and ALP Activity in Aortic Tissue of Chronic Renal Failure Rats

**Figure 1:**
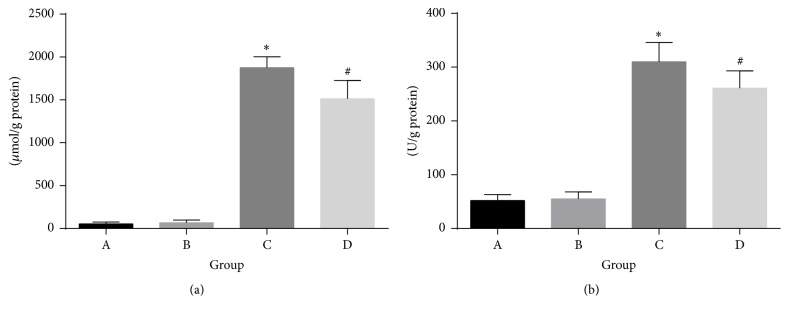
Aortic calcium accumulation (a) and ALP activity (b) in the aortic tissue. A: normal diet group; B: normal diet with quercetin supplementation group; C: adenine diet group; D: adenine diet with quercetin supplementation group. ^*∗*^*P* < 0.05 compared with A and B; ^#^*P* < 0.05 compared with A, B, and C.

After 6 weeks of treatment, the calcium in the aorta increased significantly in groups of adenine diet and adenine diet with quercetin supplementation compared with the control group, whereas the calcium in the aorta of the adenine diet with quercetin supplementation group was significantly lower than that in the adenine diet group (Figure 1(a)). Similarly, the ALP activity increased significantly in groups of adenine diet and adenine diet with quercetin supplementation compared with the control group, whereas ALP activity in the adenine diet with quercetin supplementation group was significantly lower than that in the adenine diet group (Figure 1(b)). Calcium accumulation and ALP activity in aortic tissue were militated by quercetin supplementation.

### 3.3. Histopathology of Aorta

**Figure 2:**
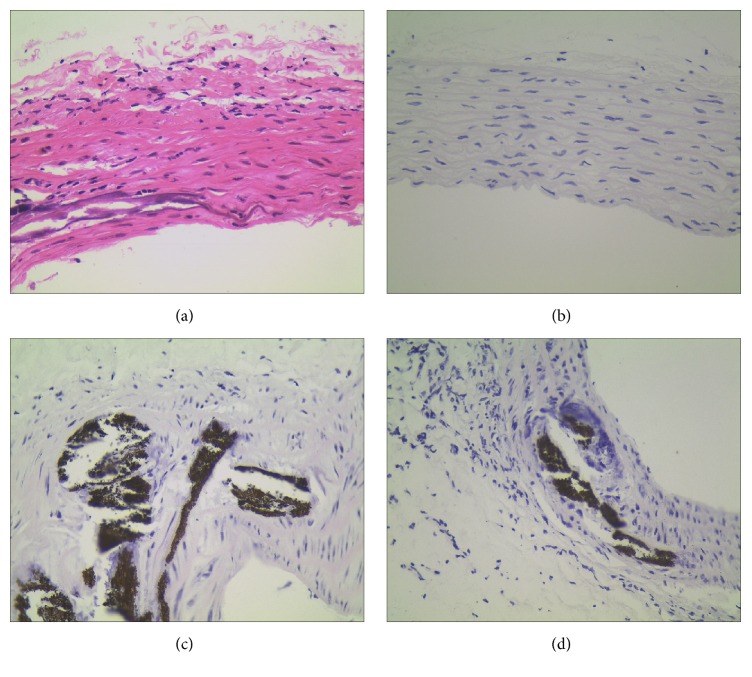
Histopathology of aorta. Control rat aorta shows normal structural arrangement (a, b), VC aorta shows patchy calcification occurring in medial layer vascular smooth muscle cell (c), and VC+ quercetin shows lower extent of calcification in medial layer vascular smooth muscle cell (d). The tissues were surgically excised and subjected to histological study by staining with Von Kossa and HE, respectively. All the magnification is 400x.

Severe calcification developed in the aorta after the treatment and the histopathology study of aorta is shown in Figure 2. Pictures of aorta exhibited that the control group and quercetin supplementation group have shown no pathogenic signature in the aortic medial layer and normal arrangement of vascular cells. However, aorta from adenine diet rats has shown patched calcification and calcium accumulation in the medial layer of vasculature. A decreased calcified region in the aorta was observed in the group of adenine diet with quercetin supplementation, which indicates that quercetin treatment prevented vascular calcification in chronic renal failure rats induced by adenine diet.

### 3.4. iNOS, p-p38MAPK, and p38MAPK Protein Expression in Aorta

**Figure 3:**
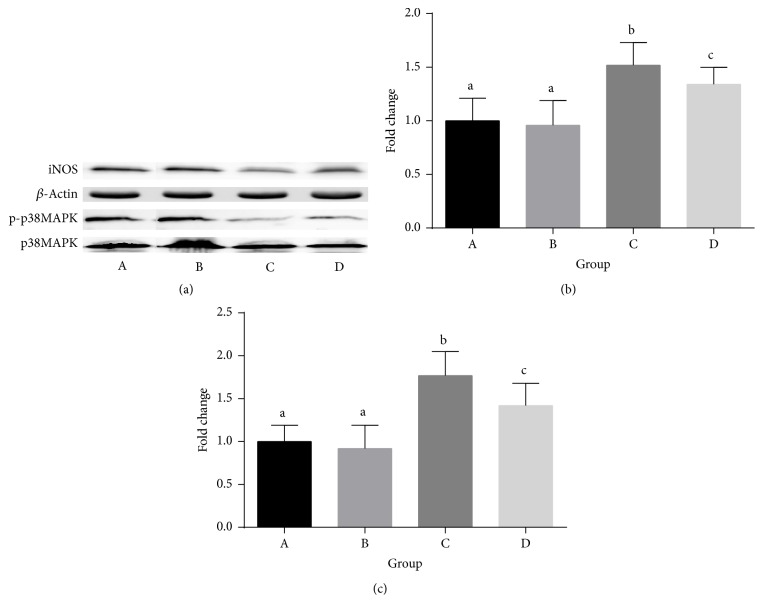
iNOS, p-p38MAPK, and p38MAPK expression in aorta. (a) Representative immunoblot showing the analysis of each protein. (b) iNOS/*β*-actin: data graphed from image densitometric analysis of blots obtained from tissues of 8 separate rats. (c) p-p38MAPK/p38MAPK: data graphed from image densitometric analysis of blots obtained from tissues of 8 separate rats. A: normal diet group; B: normal diet with quercetin supplementation group; C: adenine diet group; D: adenine diet with quercetin supplementation group. Means in the same row with different online letters differ significantly, *P* < 0.05.

Figure 3 showed that iNOS/*β*-actin ratio significantly increased in the adenine diet and adenine diet with quercetin supplementation group compared with the control group (*P* < 0.05). And iNOS/*β*-actin ratio was lower in the adenine diet with quercetin supplementation group compared with that in the adenine diet group (*P* < 0.05). p-p38MAPK/p38MAPK ratio significantly increased in the adenine diet and adenine diet with quercetin supplementation group compared with the control group (*P* < 0.05). And p-p38MAPK/p38MAPK ratio was lower in the adenine diet with quercetin supplementation group compared with that in the adenine diet group (*P* < 0.05).

## 4. Discussion

VC is a basic pathological process generally associated with atherosclerosis, hypertension, and CKD. VC is found in 80% of vascular injuries and 90% of patients with coronary artery disease [17] and is related to cardiovascular morbidity and mortality. Thus, finding agents that could inhibit VC should be a key point to prevent the cardiovascular events. The adenine-induced renal failure VC model is the most often accepted chemically induced experimental animal model for study of chronic renal failure and the relationship between oxidative stress and VC. This model has several similar mechanisms to calcification in human atherosclerosis and arteriosclerosis: high doses of adenine increased calcium content and ALP activity. Adenine is absorbed from the gastrointestinal tract and excreted by kidney. In the renal excretion process, adenine forms crystals that obstruct the renal tubules, leading to tubule interstitial fibrosis, chronic renal failure, highly extent of oxidative stress, and inflammation [18].

The present study was designed to compare the adenine-induced VC to those VC models fed with quercetin in rats. Our results revealed the significantly lower severity of VC, oxidative stress, and renal function biomarkers in quercetin group at the dose of 25 mg/kg·BW/day, compared with those in the VC group. To the best of our knowledge, this is the first investigation that found quercetin exerted a protective effect on adenine-induced VC through the modulation of oxidative stress and proteins in rats. The average daily intake of flavonoids was estimated below 100 mg and quercetin, present in foods as quercetin glycosides, represents 60–75% of the total dietary flavonols plus flavones intake [19]. This study selected only one dose of quercetin (25 mg/kg·BW). In this dose, previous studies and our research have found significant protective effects on the chronic renal failure and progression in rats [20].

Quercetin is a versatile molecular. It has been reported as a free radical scavenger and also could modulate the action of several inflammatory cytokines that are of particular concern to transplant recipients, including interleukin-1*β* (IL-1*β*), interleukin-2 (IL-2), interleukin-6 (IL-6), interleukin-15 (IL-15), and tumor necrosis factor-*α* (TNF-*α*) [21]. However, limited studies have reported the effects of quercetin on calcification in vascular cells; only two studies were related to ours. Quercetin prevented calcification of vascular smooth muscle in marginal granulocyte pool- (MGP-) null mouse model [20] and warfarin-induced calcification of vascular cells in vitro [22]. Within 6 weeks of feeding adenine-rich diet, calcification of the tunica media of the aorta was evident with Von Kossa staining. This type of calcification is independent of lipids and involves the transdifferentiation of medial smooth muscle cells into bone/cartilage-like cells with the expression of various bone-related proteins [23]. Our animal model showed VC and marked renal failure as previously described. Our study showed that quercetin ameliorated not only the renal functions, but also the VC and adenine-induced VC in rats.

Oxidative stress could lead to dysfunction of various organs, including cardiovascular diseases in CKD. Quercetin could scavenge free radicals and inhibit xanthine oxidase and lipid peroxidation. It protects against oxidative damage in renal tubular cells and renal tissues [24]. Our study showed that quercetin prevented the unfavorable changes of serum SOD and MDA in the CKD model. In line with our findings, quercetin was reported to alleviate the extent of liberation of reactive oxygen species (ROS) anti-inflammatory cytokines induced by nanozinc oxide, protected the kidneys during ischemia and reperfusion by maintaining higher levels of the enzyme xanthine dehydrogenase, and protected the cells against hydrogen peroxide induced oxidative stress and calcium dysregulation [21].

MAPK is involved in the regulation of cell proliferation, development, differentiation, apoptosis, stress, and inflammation. p38MAPK is the critical conductor of the pathway and serves as downstream effectors of ROS [25]. H_2_O_2_ activates the p38MAPK and downstream kinases in vascular smooth muscle cells of rats and human endothelial cells, resulting in heat shock protein 27 phosphorylation. p38MAPK also mediates osteogenic differentiation and calcification of calcifying vascular cells and the effects of oxidative stress in the development of VC [26]. NO is a potent vasodilator secreted primarily by endothelial cells and confers cardiovascular benefits through its action on smooth muscle [27]. And ventricular muscle and heart vascular endothelial cells of adult rats express iNOS in response to p38MAPK, which was also found to upregulate lipopolysaccharide- (LPS-) mediated iNOS expression in astrocytes and macrophages. NO could be a feedback molecular for the development of VC. It prevents the differentiation of vascular smooth muscle cells into osteoblastic cells by inhibiting TGF-beta signaling through a cGMP-dependent pathway [28]. ALP is an enzyme that has been shown to be important for matrix mineralization [29]. ALP also can promote calcification by hydrolysing pyrophosphate. Thus, inhibition of ALP activity by quercetin may suppress calcification. Our study clearly showed that increased p-P38MAPK and iNOs activity induced by adenine was effectively reversed by quercetin. Then, the inhibition of p38MAPK/iNOs pathway further reduced ALP activities and Ca levels in aorta. These indicate that quercetin prevented pathophysiological changes of VC by blocking the p38MAPK/iNOS pathway and attenuating secondary oxidative stress.

The present study has some limitations that need to be addressed. First, the anti-VC mechanism of quercetin is unclear. Whether p38MAPK/iNOs pathway is the only target of quercetin is unclear, which should be clarified in in vitro studies in the future. Second, the diverse doses of quercetin were not evaluated in the current study. Third, the results of our study cannot be transferred to human as data of other species is limited; therefore, further investigations are needed using other species or humans. However, these data provide evidence for the involvement of oxidative stress as a potential mechanism of vascular calcification, and quercetin can be treated as a potential clinical drug for prevention of vascular calcification in CKD patients.

## 5. Conclusions

In conclusion, the results of this study demonstrated, for the first time, that quercetin exerted a protective effect on vascular calcification of adenine-induced chronic renal failure rats through the modulation of oxidative stress and p38MAPK/iNOs pathway. These data provide evidence for the involvement of oxidative stress as a potential mechanism of vascular calcification and for the potential value of clinical application of quercetin to prevent vascular calcification in CKD patients. Further studies on the effects of quercetin with different doses on chronic kidney disease should be considered in humans.

## Figures and Tables

**Table 1:** Effect of quercetin on kidney function biomarkers and oxidative stress in serum.

Biomarkers	A	B	C	D
Baseline				
Body weight (g)	261.5 ± 14.6	263.8 ± 13.7	262.5 ± 15.5	261.1 ± 15.1
After intervention				
Body weight (g)	411.7 ± 31.2	401.5 ± 34.6	331.4 ± 44.6^*∗*^	341.5 ± 42.1^*∗*^

Calcium (mmol/L)	2.11 ± 0.68	2.14 ± 0.59	2.27 ± 0.57	2.23 ± 0.47
Phosphate (mmol/L)	2.12 ± 0.28	2.17 ± 0.42	5.14 ± 1.45^*∗*^	3.35 ± 0.74^*∗*,#^
Uric acid (mmol/L)	6.23 ± 0.87	7.03 ± 1.02	57.84 ± 8.81^*∗*^	44.40 ± 4.98^*∗*,#^
Creatinine (*μ*mol/L)	28.12 ± 6.98	27.89 ± 7.98	196.35 ± 23.33^*∗*^	163.54 ± 19.70^*∗*,#^
MDA (nmol/mL)	3.65 ± 0.73	3.31 ± 0.81	6.05 ± 1.06^*∗*^	4.94 ± 0.99^*∗*,#^
SOD (U/mL)	163.83 ± 14.19	166.32 ± 16.11	131.95 ± 10.25^*∗*^	146.09 ± 11.46^*∗*,#^
GSH-Px (*μ*mol/mL)	1481.50 ± 185.37	1421.78 ± 192.31	1465.25 ± 186.73	1483.38 ± 163.49

A: normal diet group; B: normal diet with quercetin supplementation group; C: adenine diet group; D: adenine diet with quercetin supplementation group; ^*∗*^*P* < 0.05 compared with A and B; ^#^*P* < 0.05 compared with C.

## References

[1] Hallan S. I., Matsushita K., Sang Y. (2012). Age and association of kidney measures with mortality and end-stage renal disease. *JAMA - Journal of the American Medical Association*.

[2] Evrard S., Delanaye P., Kamel S. (2015). Vascular calcification: from pathophysiology to biomarkers. *Clinica Chimica Acta*.

[3] Sonoki H., Sato T., Endo S. (2015). Quercetin decreases claudin-2 expression mediated by up-regulation of microRNA miR-16 in lung adenocarcinoma A549 cells. *Nutrients*.

[4] Yan S., Wu B., Lin Z. (2009). Metabonomic characterization of aging and investigation on the anti-aging effects of total flavones of Epimedium. *Molecular BioSystems*.

[5] Guo X. D., Zhang D., Gao X. (2013). Quercetin and quercetin-3-O-glucuronide are equally effective in ameliorating endothelial insulin resistance through inhibition of reactive oxygen species-associated inflammation. *Molecular Nutrition and Food Research*.

[6] Lamson D. W., Brignall M. S. (2000). Antioxidants and cancer III: quercetin. *Alternative Medicine Review*.

[7] Papiez M. A., Cierniak A., Krzysciak W. (2008). The changes of antioxidant defense system caused by quercetin administration do not lead to DNA damage and apoptosis in the spleen and bone marrow cells of rats. *Food and Chemical Toxicology*.

[8] Nishimuro H., Ohnishi H., Sato M. (2015). Estimated daily intake and seasonal food sources of quercetin in Japan. *Nutrients*.

[9] Serban M., Sahebkar A., Zanchetti A. (2016). Effects of quercetin on blood pressure: a systematic review and meta‐analysis of randomized controlled trials. *Journal of the American Heart Association*.

[10] Huang R., Zhong T., Wu H. (2015). Quercetin protects against lipopolysaccharide-induced acute lung injury in rats through suppression of inflammation and oxidative stress. *Archives of Medical Science*.

[11] Song Y., Liu J., Zhang F., Zhang J., Shi T., Zeng Z. (2013). Antioxidant effect of quercetin against acute spinal cord injury in rats and its correlation with the p38MAPK/iNOS signaling pathway. *Life Sciences*.

[12] Franczyk-Skóra B., Gluba A., Banach M., Rozentryt P., Poloński L., Rysz J. (2013). Acute coronary syndromes in patients with chronic kidney disease. *Current Vascular Pharmacology*.

[13] Gomes I. B. S., Porto M. L., Santos M. C. L. F. S. (2014). Renoprotective, anti-oxidative and anti-apoptotic effects of oral low-dose quercetin in the C57BL/6J model of diabetic nephropathy. *Lipids in Health and Disease*.

[14] Chaudhary S., Ganjoo P., Raiusddin S., Parvez S. (2014). Nephroprotective activities of quercetin with potential relevance to oxidative stress induced by valproic acid. *Protoplasma*.

[15] Manivannan J., Barathkumar T. R., Sivasubramanian J., Arunagiri P., Raja B., Balamurugan E. (2013). Diosgenin attenuates vascular calcification in chronic renal failure rats. *Molecular and Cellular Biochemistry*.

[16] Cai D.-Y., Yu F., Jiang W. (2005). Adrenomedullin(27-52) inhibits vascular calcification in rats. *Regulatory Peptides*.

[17] Demer L. L., Tintut Y. (2008). Vascular calcification: pathobiology of a multifaceted disease. *Circulation*.

[18] Ali B. H., Al-Husseni I., Beegam S. (2013). Effect of gum arabic on oxidative stress and inflammation in adenine-induced chronic renal failure in rats. *PLoS ONE*.

[19] Perez-Vizcaino F., Duarte J., Jimenez R., Santos-Buelga C., Osuna A. (2009). Antihypertensive effects of the flavonoid quercetin. *Pharmacological Reports*.

[20] Beazley K. E., Lima F., Borras T., Nurminskaya M. (2013). Attenuation of chondrogenic transformation in vascular smooth muscle by dietary quercetin in the MGP-deficient mouse model. *PLoS ONE*.

[21] Faddah L. M., Abdel Baky N. A., Al-Rasheed N. M., Al-Rasheed N. M., Fatani A. J., Atteya M. (2012). Role of quercetin and arginine in ameliorating nano zinc oxide-induced nephrotoxicity in rats. *BMC Complementary and Alternative Medicine*.

[22] Beazley K. E., Eghtesad S., Nurminskaya M. V. (2013). Quercetin attenuates warfarin-induced vascular calcification in vitro independently from matrix gla protein. *Journal of Biological Chemistry*.

[23] Mathew S., Tustison K. S., Sugatani T., Chaudhary L. R., Rifas L., Hruska K. A. (2008). The mechanism of phosphorus as a cardiovascular risk factor in CKD. *Journal of the American Society of Nephrology*.

[24] Al-Rasheed N. M., Faddah L. M., Mohamed A. M., Abdel Baky N. A., Al-Rasheed N. M., Mohammad R. A. (2013). Potential impact of quercetin and idebenone against immuno- inflammatory and oxidative renal damage induced in rats by titanium dioxide nanoparticles toxicity. *Journal of Oleo Science*.

[25] Tanikawa T., Okada Y., Tanikawa R., Tanaka Y. (2009). Advanced glycation end products induce calcification of vascular smooth muscle cells through rage/p38 MAPK. *Journal of Vascular Research*.

[26] Kang J.-H., Toita R., Asai D., Yamaoka T., Murata M. (2013). Reduction of inorganic phosphate-induced human smooth muscle cells calcification by inhibition of protein kinase A and p38 mitogen-activated protein kinase. *Heart and Vessels*.

[27] Behrendt D., Ganz P. (2002). Endothelial function. *The American Journal of Cardiology*.

[28] Kanno Y., Into T., Lowenstein C. J., Matsushita K. (2008). Nitric oxide regulates vascular calcification by interfering with TGF-*β* signalling. *Cardiovascular Research*.

[29] Stein G. S., Lian J. B., Stein J. L., Van Wijnen A. J., Montecino M. (1996). Transcriptional control of osteoblast growth and differentiation. *Physiological Reviews*.

